# A quantitative assessment of the content of hematopoietic stem cells in mouse and human endosteal-bone marrow: a simple and rapid method for the isolation of mouse central bone marrow

**DOI:** 10.1186/s12878-015-0031-7

**Published:** 2015-07-09

**Authors:** Maya M. Mahajan, Betty Cheng, Ashley I. Beyer, Usha S. Mulvaney, Matt B. Wilkinson, Marina E. Fomin, Marcus O. Muench

**Affiliations:** Blood Systems Research Institute, 270 Masonic Ave., San Francisco, CA USA; Department of Laboratory Medicine, University of California, San Francisco, CA USA

**Keywords:** Hematopoietic stem cells, Bone marrow cells, Cell culture techniques, Cell count, Stem cell niche, Flow cytometry, Mice, Humans, Transplantation, Chimera

## Abstract

**Background:**

Isolation of bone marrow cells, including hematopoietic stem cells, is a commonly used technique in both the research and clinical settings. A quantitative and qualitative assessment of cell populations isolated from mouse and human bone marrow was undertaken with a focus on the distribution of hematopoietic cells between the central bone marrow (cBM) and endosteal bone marrow (eBM).

**Methods:**

Two approaches to cBM isolation from the hind legs were compared using the C57BL/6J and BALB/cJ strains of laboratory mice. The content of hematopoietic stem cells in eBM was compared to cBM from mice and human fetal bone marrow using flow cytometry. Enzymatic digestion was used to isolate eBM and its effects on antigen expression was evaluated using flow cytometry. Humanized immunodeficient mice were used to evaluate the engraftment of human precursors in the cBM and eBM and the effects of in vivo maturation on the fetal stem cell phenotype were determined.

**Results:**

The two methods of mouse cBM isolation yielded similar numbers of cells from the femur, but the faster single-cut method recovered more cells from the tibia. Isolation of eBM increased the yield of mouse and human stem cells. Enzymatic digestion used to isolate eBM did, however, have a detrimental effect on detecting the expression of the human HSC-antigens CD4, CD90 and CD93, whereas CD34, CD38, CD133 and HLA-DR were unaffected. Human fetal HSCs were capable of engrafting the eBM of immunodeficient mice and their pattern of CD13, CD33 and HLA-DR expression partially changed to an adult pattern of expression about 1 year after transplantation.

**Conclusions:**

A simple, rapid and efficient method for the isolation of cBM from the femora and tibiae of mice is detailed. Harvest of tibial cBM yielded about half as many cells as from the femora, representing 6.4 % and 13 %, respectively, of the total cBM of a mouse based on our analysis and a review of the literature. HSC populations were enriched within the eBM and the yield of HSCs from the mouse and human long bones was increased notably by harvest of eBM.

**Electronic supplementary material:**

The online version of this article (doi:10.1186/s12878-015-0031-7) contains supplementary material, which is available to authorized users.

## Background

Collection of bone marrow (BM) from mice is an integral part of a broad range of studies in the fields of hematology and immunology. Murine BM is also a source of other cell types such as mesenchymal stromal cells (MSCs), endothelial cells, osteoblasts, and osteoclasts [1–4]. BM samples are most typically obtained from femora and sometimes tibiae. The method of isolating BM cells typically involves cleaning some degree of soft-tissue from the bone and flushing cells out of the marrow cavity using a syringe with a fine needle [1]. However, based on descriptions in the literature and our own research team’s experiences, there are a number of different approaches to the isolation of BM from mouse limb bones. The main difference in approach is whether investigators choose to flush marrow from the bones by removal of one [5] or both epiphyses [1]. Additionally, investigators differ on the degree of soft tissue removal performed prior to flushing the bones. Extensive removal of soft-tissue can be a time-consuming process with an uncertain benefit on the yield of BM cells.

The harvest of BM from human bone samples obtained after surgery from living donors or from cadavers is an important source of tissue for research [6] and may also have clinical use [7]. For instance, BM harvested from the long bones of fetal specimens has been used as a source of hematopoietic stem cells (HSCs) [8] and MSCs [9, 10] for research. These cells have also been proposed as a source of donor cells for clinical transplantation [11–13].

The distribution of cell types within the BM is not homogeneous and, consequently, different harvest techniques may vary in their efficiency in isolating particular cell lineages [14]. Studies of the stem cell niche have shown different types of stem cells and progenitors to reside in different parts of the long-bone marrow. Lord and Hendry were among the first to show an increased density of hematopoietic precursors with distance away from the central axis of the bone – referred to as the central bone marrow (cBM) [15]. Accordingly, higher levels of precursor proliferation are found near the inner wall of the bone, closer to the endosteum, the location of the endosteal bone marrow (eBM) [16].

Recently, Grassinger et al. demonstrated that phenotypically defined HSCs were enriched within the eBM of the mouse [17]. These authors estimated that about a quarter of all CD48^−^CD150^+^CD117^+^SCA-1^+^ lineage (Lin)-depleted HSCs reside in the endosteum. Likewise, human HSCs are enriched in the trabecular bone found at the ends of the long bones [18]. In chimeric mice, created by the transplantation of human HSCs into immunodeficient mice [19], human HSCs are preferentially localized to the eBM in the metaphysis and epiphysis [18]. Similar to the findings on human bones, transplanted human HSCs were enriched in the trabeculae of the metaphysis/epiphysis of the murine femur. To note, harvest of eBM is technically more time-consuming and costly than simply flushing or rinsing BM from bones as it involves removal of soft tissue, crushing of the bone and enzymatic digestion [20].

One objective in performing this study was to discern the most reliable, simplest and fastest method for the routine isolation of cBM. Two methods were compared: in one method, cBM was flushed from an incision at one end of the bone after only minimal removal of soft tissue and, in a second method, cBM was harvested following removal of the majority of soft tissue and flushing the cBM from one end of the bone and out the other end. These two procedures are referred to simply as the single-cut and double-cut methods, respectively. Both methods were tested on the femora and tibiae of mice and the number of cells obtained and the time required for harvest was compared. In addition, researchers with varying degrees of experience with these procedures were studied to gauge the difficulty in learning the two techniques. Herein, we also quantified the enzymatic harvest of eBM to compare the efficacy of recovering HSCs by this method compared to simple flushing methods used to collect cBM. We not only evaluated the benefit of harvesting eBM for the collection of murine HSCs, but also human HSCs isolated from fetal BM and humanized immunodeficient mice. Lastly, we compared the phenotypic profile of human HSCs isolated from the cBM and eBM from both fetal BM and from transplanted fetal cells recovered from humanized mice.

## Methods

### Mice

This study was conducted with approval of the Institutional Animal Care and Use Committee at ISIS Services LLC (San Carlos, CA). The C57BL/6J and BALB/cJ strains were purchased from The Jackson Laboratories (Bar Harbor, MN and Sacramento, CA). Breeder pairs of immunodeficient NOD.Cg-*Prkdc*^*scid*^*Il2rg*^*tm1Wjl*^/SzJ (NSG) mice were also obtained from The Jackson Laboratories and bred at our institute. Additionally, NOD.Cg-*Prkdc*^*scid*^*Il2rg*^*tm1Sug*^*Tg*(*Alb*-*Plau*)*11*-*4*/ShiJic (uPA-NOG) mice were bred at our institute by crossing uPA transgene hemizygous X homozygous mice [21].

Male and female mice were obtained through tissue sharing whenever possible and killed by orbital enucleation and exsanguination or by CO_2_ asphyxiation and cervical dislocation. All animals were adults (≥8 weeks of age) at the time of sacrifice. Mice were maintained in microisolator cages in a facility free of commonly tested murine pathogens and received humane care according to the criteria outlined by the National Research Council’s Institute of Laboratory Animal Resources in the “Guide for the Care and Use of Laboratory Animals”.

### Human tissues

Human fetal long-bones and liver were collected from San Francisco General Hospital with consent from the mothers undergoing elective abortions and with approval of the University of California San Francisco’s Committee on Human Research. The gestational age of the tissues used was between 20–24 weeks old estimated based on the foot length of the fetus. All specimens were donated anonymously.

### Harvest of mouse cBM

**Fig. 1:**
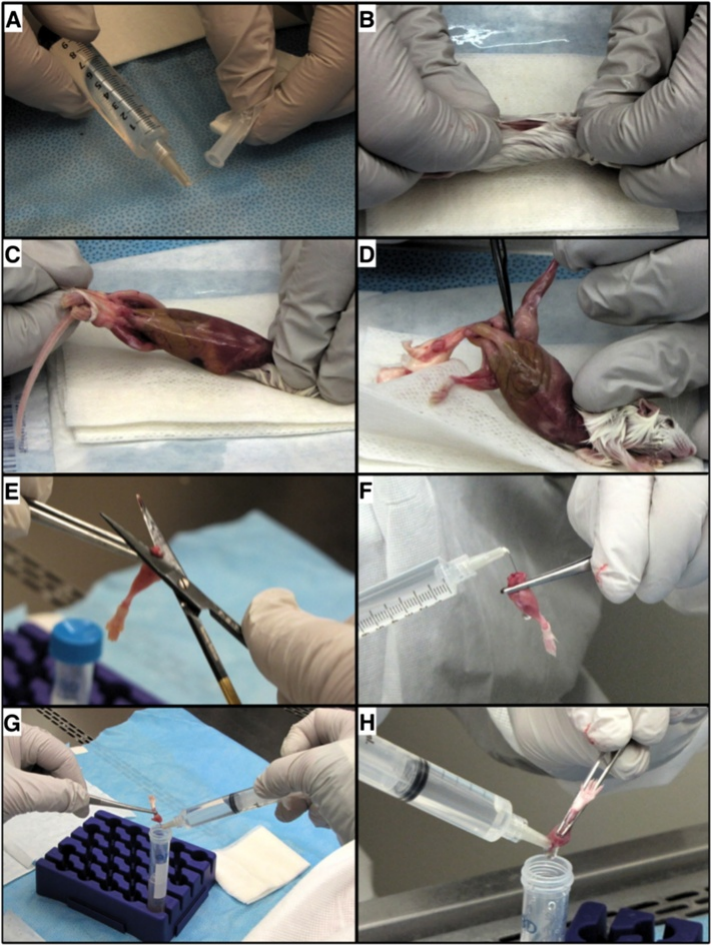
Stepwise overview of the single-cut method for mouse cBM isolation. Panels (**a**)-(**h**) demonstrate the key steps in the isolation of cBM. Refer to the protocol text for an explanation of the individual steps

**Fig. 2:**
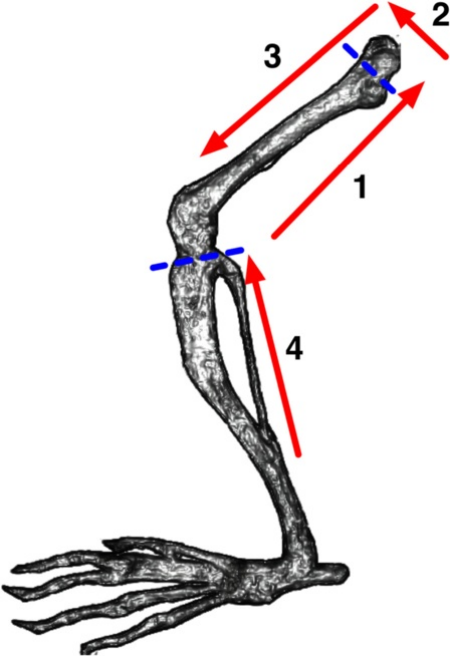
Stepwise schematic of cuts made in soft-tissue and bone for the isolation of mouse cBM. The direction and numerical sequence of soft-tissue cuts are shown using red arrows. The approximate location of cuts made to remove the bone epiphyses are shown with dashed blue lines

The following supplies are required to harvest cBM from mice: 70 % ethanol, phosphate-buffered saline (PBS), 50 ml and 15 ml tubes, sterile gauze pads, a 10 ml syringe, needles, forceps, and scissors. Unless otherwise stated, 27 gauge, 13 mm needles (Becton Dickinson, Franklin Lakes, NJ) were used in this study, but testing of different needle gauges indicate 23 gauge needles offer the best performance. A video has been posted online (see Additional file 1) demonstrating the single-cut method of isolating cBM, which was made following this protocol: Fill a 10 mL syringe with PBS using a 50 ml tube as a reservoir. Attach a 23 gauge, 19 mm needle (Becton Dickinson, Franklin Lakes, NJ) and bend 90° using the plastic cap (Fig. 1a). After sacrifice, spray the mouse with 70 % ethanol to wet the fur to prevent its dispersal before removal of the skin. Make a small incision in the skin above the abdomen of the mouse (Fig. 1b). From this cut, pull the skin apart and gently remove the legs, one at a time, from the skin (Fig. 1c). The legs are removed in the following manner: Bend back the leg dorsally as if to dislocate the joint from the pelvis. While pulling the leg, use scissors to make three cuts to detach the leg from the pelvis (Fig. 1d). The first cut is roughly parallel to the femur towards the pelvis (Fig. 2), thereby removing soft tissue from the femur while severing some of the soft-tissue that connects the femur to the hip. The second cut is made while dislocating the femur to reveal the proximal epiphysis of the femur. The third cut, similar to the first, is made parallel to the femur, towards the knee, to further reduce the soft-tissue around the femur and detach the leg. To collect the cBM from the femur, remove the proximal epiphysis with scissors (Fig. 1e). To reduce the loss of BM, it is important to cut just below the ball joint (Fig. 2). The red BM should be visible. Insert the needle into the opening of the bone (Fig. 1f). Flush the cBM cells into a collection tube by injecting approximately 0.5-3 mL of PBS while holding the bone over the opening of the tube. For the tibia, remove the bulk of the soft tissue by holding the tibia using forceps and cutting roughly parallel towards the knee (Fig. 2). Cut the proximal epiphysis of the tibia by cutting just below the knee joint (Fig. 1). The white ligaments covering the knee provide a landmark and the cut should be immediately below this connective tissue. Flush cells from the tibia as for the femur (Fig. 1g-h).

The double-cut method differed from the single-cut method in that the bone was first cleaned of the majority of soft tissue to allow for visualization of the bones. Complete soft-tissue removal, as is sometimes performed when isolating cBM [1], was not done to allow the duration of the two isolation methods to be more reasonably compared. Then, both the distal and proximal epiphyses were removed and marrow was flushed from the distal out the proximal end of the bones.

For both the single-cut and double-cut methods, the volume of PBS used to harvest the cBM was not fixed. The mean volume used to flush femora was 2.2 ml and 2.0 ml using the single-cut and double-cut methods, respectively (n = 56). For the tibiae, an average of 1.8 ml and 1.6 ml were used with the single-cut and double-cut methods, respectively.

The time required to harvest the cBM by the single-cut and double-cut methods was recorded starting at the time of the excision of the leg. Data were collected starting on the left leg and the method of harvest used was alternated between the left and right legs to account for the handedness of the investigators. This approach was used to balance the number of cBM cells recovered and the time required to perform the two procedures. Although the procedure was timed, the investigators were instructed to primarily focus on an accurate collection of the most cells possible with the speed of the procedure being of secondary concern.

### Isolation of mouse eBM

Murine eBM was harvested in a manner similar to a previously described method [20]. Briefly, femurs were removed of all soft tissue using scissors and forceps. Bones were cut into smaller fragments and digested with 3 mg/ml collagenase I (Sigma Aldrich, St. Louis, MO) and 4 mg/ml dispase II (Roche Diagnostics Corporation, Indianapolis, IN) at 37 °C for 5 minutes. The eBM was filtered through a 40 μm strainer and washed once by centrifugation.

### Isolation of fetal human cBM and eBM

Human cBM was isolated from either a femur or all long bones as previously detailed [12]. Briefly, bones were denuded of all soft tissue, including the fibrous periosteum, using a scalpel and scissors. cBM was flushed with a 19 gauge needle (Becton Dickinson) inserted, often at multiple sites, into both ends of the bone and flushed with PBS until the red-cell content of the marrow was visibly depleted. PBS was used to rinse the bones. Each bone, immersed in fresh PBS, was then cut in half lengthways and the cBM scraped from the bone with a scalpel blade and filtered through a 100 μm cell strainer (Greiner Bio-One, Germany). The cBM was washed once by centrifugation.

The bone fragments that remained after flushing and filtration of the cBM were enzymatically digested to isolate eBM as described for the mouse bones. The eBM was filtered and washed once by centrifugation before flow cytometric analysis.

### Construction of humanized mice

Male and female mice were transplanted with human hematopoietic cells to establish hematopoietic chimeras. Immunodeficient uPA-NOG mice [21] were transplanted with fetal liver cells, prepared as described [22]. Cells were transplanted by intra-splenic injection without prior cytoablative irradiation [23]. Additionally, NSG mice were transplanted intravenously with 2 × 10^6^ midgestation light-density cBM cells after 175 cGy X-ray irradiation.

### Flow cytometric analysis

Human BM cells were suspended in blocking buffer consisting of PBS with 5 % mouse serum and 0.01 % NaN_3_ and, for samples containing mouse BM, the blocking buffer was supplemented with 2 μg/ml rat anti-mouse CD16/CD32 mAb (BioLegend, San Diego, CA). Samples were stained with monoclonal antibodies and live cells, identified using propidium iodide staining, analyzed on a flow cytometer as previously described [24]. Data were analyzed using FlowJo software, version 9.7 (Tree Star, Inc.; Ashland, OR).

The following fluorescein isothiocyanate (FITC)-labelled, phycoerythrin (PE)-labelled, allophycocyanin (APC), PE-cyanine 7 (PE-Cy7), APC-cyanine 7 (APC-Cy7), pacific blue (PB) or Alexa Fluor 700 (AF700) antibodies were purchased from BioLegend or an otherwise stated vendor: mouse IgG1 FITC, mouse IgG1 PE, mouse IgG1 APC, mouse IgG1 PE-Cy7, mouse IgG1 APC-Cy7 and mouse IgG2a PE. The following monoclonal antibodies were used to stain human cells: CD45 FITC (clone HI30), CD45 APC-Cy7 (clone HI30), FITC-labeled mature lineage cocktail (clones UCHT1, HCD14, 3G8, HIB19, 2H7, HCD56), CD133 APC (clone AC133; Miltenyi Biotec, Auburn, CA), CD133 PE (clone AC133; Miltenyi Biotec), CD34 PE-Cy7 (clone 581), CD4 PE (clone L200; BD Pharmingen, San Diego CA), CD13 APC (clone WM15; BD Pharmingen), CD33 (clone WM53), CD38 PE (clone HIT2), CD90 PE (clone 5E10), CD93 PE (clone VIMD2), CD147 (clone TRA-1-85; R&D Systems, Minneapolis, MN), HLA-DR FITC (clone L243; Becton Dickinson) and HLA-DR PE (clone L243). The following monoclonal antibodies were used to stain mouse cells: CD3 (clone17A2), CD11b (clone M1/70), CD45R (clone RA3-6B2), Gr-1 (clone RB6-8C5), TER-119 (clone TER-119), CD45 (clone 30-F11), H-2K^d^ (clone SF1-1.1) all PB labelled, CD48 PE-Cy7 (clone HM48-1), CD117 (c-kit) FITC (clone 2B8), CD150 (SLAM) PE (clone TC15-12 F12.2) and SCA-1 APC (clone D7).

### Cell counts

Live cell counts were performed using a Scepter Handheld Automated Cell Counter with 40 μm sensors (EMD Millipore Corporation, Billerica, MA, USA). Particles >3 μm were counted.

### Data presentation and statistical analysis

Statistical analysis and charting was performed using Aabel 3 and Aabel NG software (Gigawiz Ltd. Co. OK, USA). The 2-tailed Wilcoxon Matched-Pairs test was used to determine the significance of differences between cell yields obtained by the single-cut and double-cut methods. The Mann–Whitney U-test was used to determine the significance of differences in cell population frequencies between cBM and eBM. Differences were considered significantly different at P ≤ 0.05. Notched box and whisker plots are used to display some of the data where the box represents the distribution of the 25th through 75th percentile of the data and the whiskers extend to the extreme data points. Medians are represented by the notch in the box.

## Results

### Comparison of the single-cut and double-cut methods of mouse cBM isolation

**Fig. 3:**
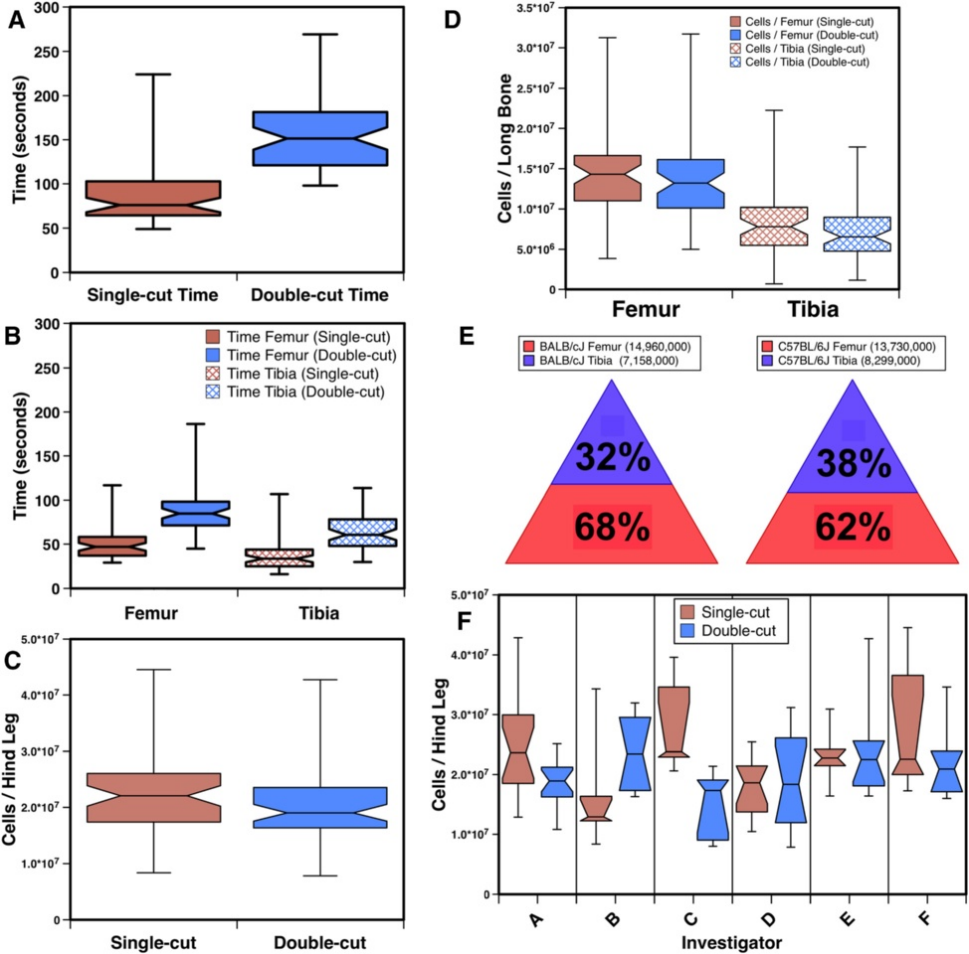
Comparisons of mouse cBM isolations using the single-cut and double-cut methods. The time required to isolate cBM by the two methods is shown for both the femur and tibia (**a**) and for each of the long bones individually (**b**). The number of cells isolated by the two methods from both long-bones is shown in (**c**) and cell yields for the individual bones are shown in (**d**). The percentages of tibial and femoral BM cells recovered from BALB/cJ (n = 19) and C57BL/6J (n = 37) mice are shown (**e**). The median number of cells recovered from each bone using the single-cut method are indicated in the legends. Cell yields are shown by investigator in (**f**). Refer to Table 1 for the number of harvests performed and the level of experience of the investigator

Two methods of mouse cBM isolation were tested. Investigators with differing levels of experience with the procedures performed these experiments to determine which method was best for experienced and novice investigators (Table 1). The two methods were compared to determine which was the most reliable and/or fastest. The median time using the single-cut method was much faster (76 seconds, n = 56) than the double-cut method (151.5 seconds, n = 56) regardless of experience level of the investigator (Fig. 3a and Table 1). Not surprisingly, investigators experienced with the single-cut method were able to perform their isolations most rapidly (Table 1). Moreover, the single-cut method was the fastest method for isolating cBM from both the femora and tibiae (Fig. 3b).

**Table 1:** Summary of investigator experience and cBM collection times

Investigator	Years experience	Preferred method	Number of harvests	Median time (Seconds) single-cut method	Median time (Seconds) double-cut method
A	≥25 years	Single-cut	18	62	120
B	≥25 years	Double-cut	5	98	187
C	≥5 years	Single-cut	5	68	170
D	<1 year	Novice	12	88	174
E	<1 year	Novice	10	121	149
F	<1 year	Novice	6	75	117

The median number of cells harvested by the single-cut method was 2.21 × 10^7^, compared to 1.91 × 10^7^ obtained by the double cut method (n = 56, Fig. 3c). The 15.7 % increased yield of cBM obtained by the single-cut method was not significant (*P* = 0.057). When the cell yields were analyzed separately for the two hind-leg bones a modest 8.2 % higher number of femoral cells were recovered using the single-cut method (Fig. 3d), which was not significantly higher than the yield obtained with the double-cut method. However, the 19.4 % increased recovery of cells from the tibia using the single-cut method was significant (*P* = 0.043). Thus, the single-cut method is a favorable method for isolating cBM from both the femur and the tibia.

Given that the murine tibia is a smaller bone than the femur and extra effort is required to harvest cells from it, we sought to determine the quantity of cBM that could be harvested from the tibia. The results were very similar for two strains of mice analyzed (Fig. 3e). For both strains combined, the tibia yielded about 54 % the number of cells obtained from the femur. Stated in another way, about a third of the cBM available for harvest from a hind leg is found in the tibia.

We evaluated the cell yields individually by investigator to gain insight into the effects of experience on performing the two procedures. There was no significant difference in cell yield between the combined results of the 3 experienced investigators and 3 novice investigators (n = 28 harvests each group) for either of the two isolation methods. Investigators A and C, both experienced with the single-cut method, did recover a significantly higher number of cells using this method (Fig. 3f), whereas investigator B, previously experienced with the double-cut method, obtained an insignificantly greater number of cells with their familiar procedure. There was no significant difference in cell yield among the novice investigators comparing the two isolation methods.

### Comparison of the yields of hematopoietic precursors found in mouse cBM and eBM

**Fig. 4:**
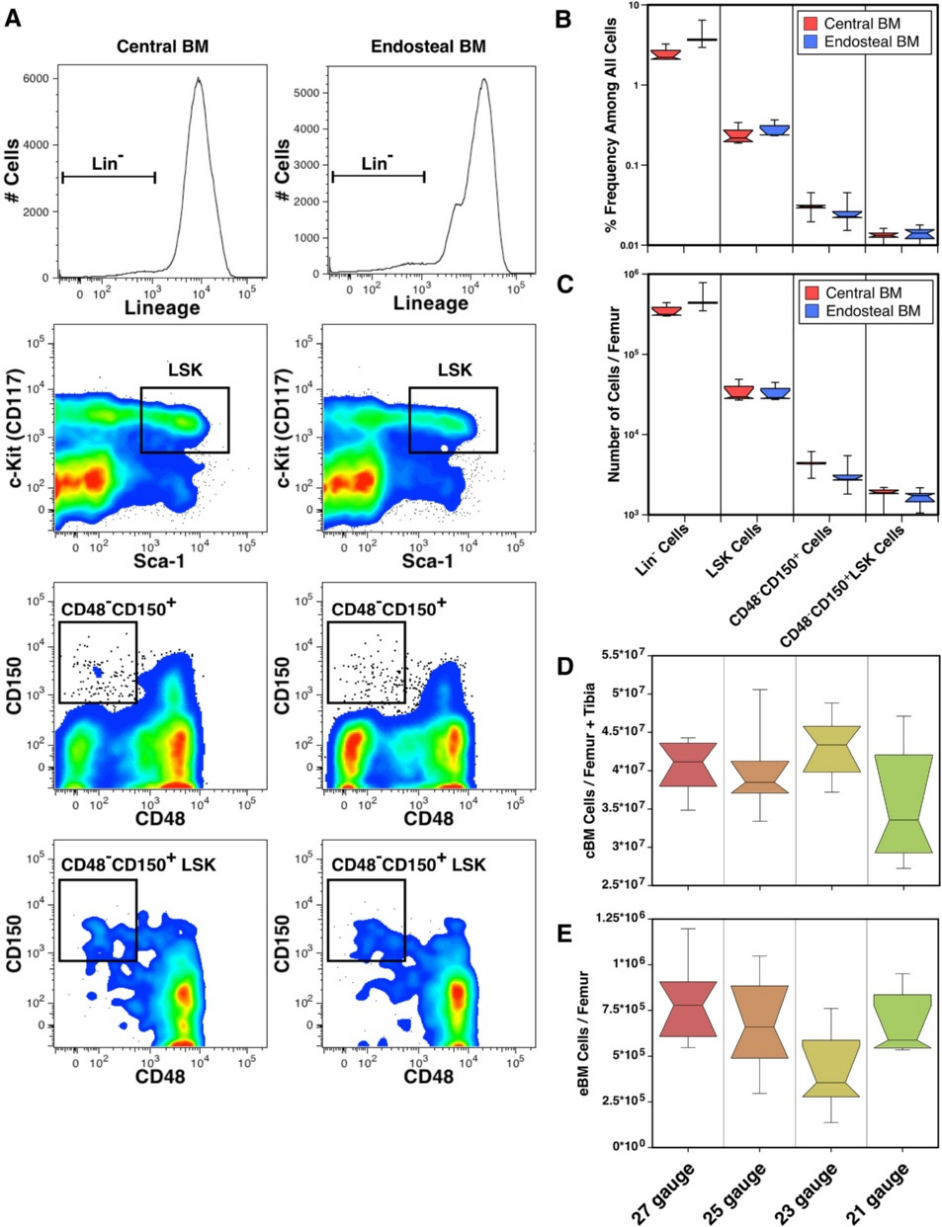
The quantities of C57BL/6J mouse hematopoietic precursors from cBM and eBM. Flow cytometric analysis of cBM and eBM was used to identify precursor populations using the gates indicated (**a**). Total cells were defined as live cells – lacking PI staining – from which doublets were excluded by electronic gating (not shown). The median frequencies (**b**) and numbers (**c**) of cells recovered of each precursor population are shown (n = 5). Note, that these data are shown on a logarithmic scale. The effects of needle size on the recovery of cBM (**d**) and eBM (**e**) cells are shown for 6 samples for each group

We sought to quantify the number of cells and, more specifically, hematopoietic precursors that remained in the bones after flushing out cBM using the single-cut method. Isolation of eBM recovered 84 % more cells than by flushing alone. The yields of different hematopoietic progenitor compartments were determined using flow cytometric analysis (Fig. 4a). The frequency (Fig. 4b) and total number (Fig. 4c) of Lin^−^ cells recovered from the femurs of 5 mice were greater from the eBM than the cBM. Recoveries of Lin^−^SCA-1^+^CD117^+^ (LSK) cells, Lin^−^CD48^−^CD150^+^ cells and CD48^−^CD150^+^ LSK cells did not differ significantly between the cBM and eBM. These data indicate that approximately equivalent numbers of progenitors and HSCs can be recovered from the cBM and eBM if flushed with a small 27 gauge needle size.

As the size of needle used to flush cBM from the long bones may affect the recovery of cBM and eBM. We compared harvests of cBM, from the femur and tibia, using four needle sizes (Fig. 4d). There were no significant differences in the yield of cBM using the different gauged needles. There was, however, a more varied and decreased median recovery using the largest needles (21 gauge). These needles were too large to easily insert in the marrow cavities of tibiae resulting, sometimes, in fractures. Indeed, there was a significant decrease in the harvest of tibial cBM using 21 gauge needles compared to 25 and 27 gauge needles (*P* = 0.016 and 0.007, respectively), which affected the overall yield of cBM. The recovery of the remaining eBM was less using 23 gauge needles than using the smaller 27 gauge needles (Fig. 4e, *P* = 0.025), which was mirrored by an increased recovery of femoral cBM (not shown, *P* = 0.037). Using 23 gauge needles, the recovery of eBM was only 1.1 % of cBM. Thus, increasing the needle size up to 23 gauge can modestly increase the recovery of cBM and reduce the amount BM cells left in the femur. This affect wasn’t apparent using the even larger 21 gauge needles likely because the tight fit of these needles within the marrow cavity affected the ability to flush the bones.

### Comparison of hematopoietic precursors yields from fetal human cBM and eBM

**Fig. 5:**
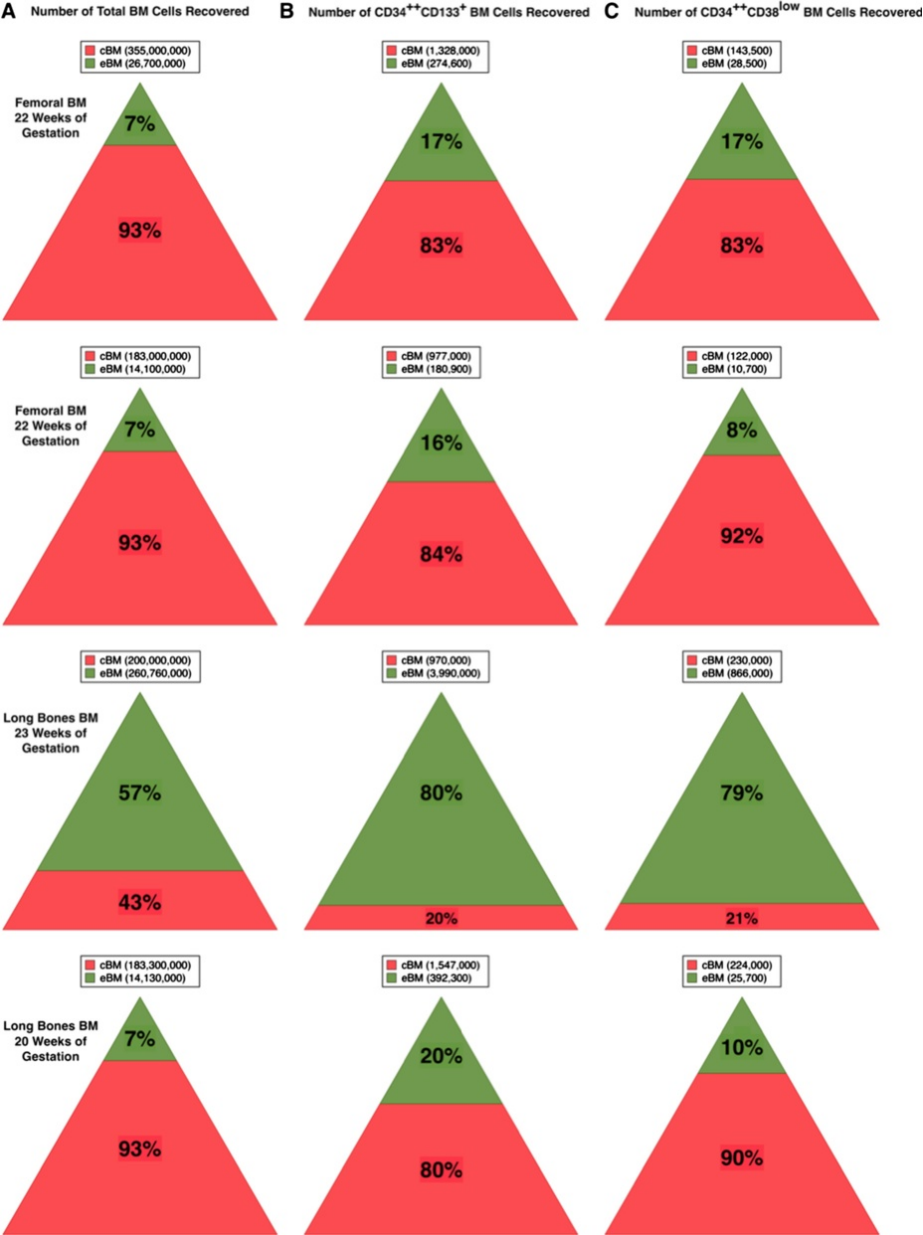
Isolation of human hematopoietic precursors from cBM and eBM. The relative recovery of total cells (**a**), CD34^++^CD133^+^ cells (**b**) and CD34^++^CD38^low^ cells (**c**) from cBM and eBM are shown graphically for 4 samples. The number of cells recovered are indicated in the legends above each triangle

The eBM compartment was also examined in human midgestation long bones. Four specimens of fetal human long-bones were used to isolate cBM and eBM and the recovered cells were analyzed by flow cytometry to determine the yield of HSCs as well as other BM cell populations. In 3 of 4 experiments, eBM cells represented a consistent 7 % of all BM cells recovered (Fig. 5). However, in one experiment, 57 % of cells were recovered from the eBM fraction. We attribute this outlier to the older age of the specimen and the fact that all the long bones found in the legs and arms were processed instead of a single femur as in the first two experiments. The greater amount of tissue processed and hardness of the bones most likely led to less efficient flushing of the cBM, thereby resulting in a greater yield in the subsequent enzymatic digestion of the tissue.

**Fig. 6:**
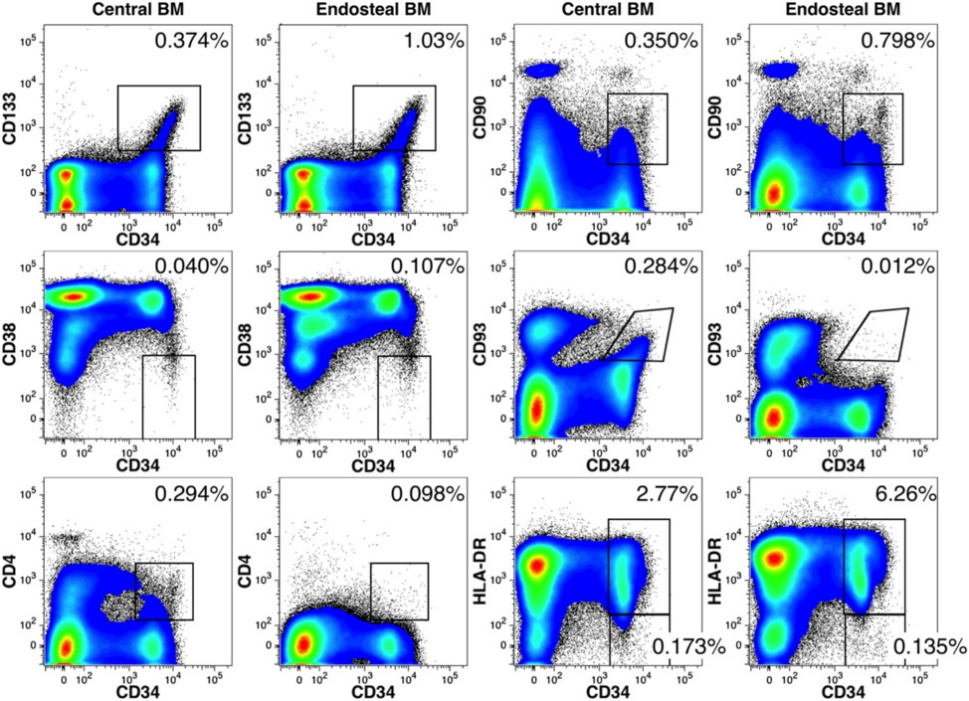
Flow cytometric analysis of fetal human cBM and eBM. Side-by-side comparisons of cell surface markers on CD34^++^ cells. The frequencies of gated cell populations among live-single cells are indicated in each plot. In addition to the markers shown, hematopoietic precursors were also defined by their expression of CD45 and light-scatter gate that excluded high side-light scatter events (not shown). Femoral cells were obtained from a 22 weeks’ gestation fetus

To estimate the distribution of HSCs between the cBM and eBM compartments of the fetal long bones, two cell populations enriched in HSCs were enumerated from the BM preparations: CD34^++^CD133^+^ and CD34^++^CD38^low^ cells (Fig. 5). In all four samples, the frequencies of these primitive progenitors were enriched among eBM cells. CD34^++^CD38^low^ cells represent a small subset of CD34^++^CD133^+^ cells and their yield was noticeably lower than for CD34^++^CD133^+^ cells. A phenotypic profile using a number of markers associated with HSCs further demonstrates the enrichment of HSCs and primitive progenitors among the eBM fraction of BM cells (Fig. 6). The representative analysis shows the enrichment of CD34^++^CD133^+^ and CD34^++^CD38^low^ cells found in the eBM fraction. Likewise, CD34^++^CD90^+^ cells, representing another population enriched in stem cells [25], was also enriched more than 2-fold among eBM cells. In fetal tissues, most primitive progenitors express HLA-DR, although some are HLA-DR^low/-^ [26, 27], whereas adult cells with the properties of HSCs are enriched among HLA-DR^−^ cells [28–30]. CD34^++^HLA-DR^+^ cells were enriched among eBM cells, whereas the frequency of CD34^++^HLA-DR^−^ cells was more or less similar in the two BM preparations. Overall, an enrichment of primitive progenitor populations was observed among eBM cells.

**Fig. 7:**
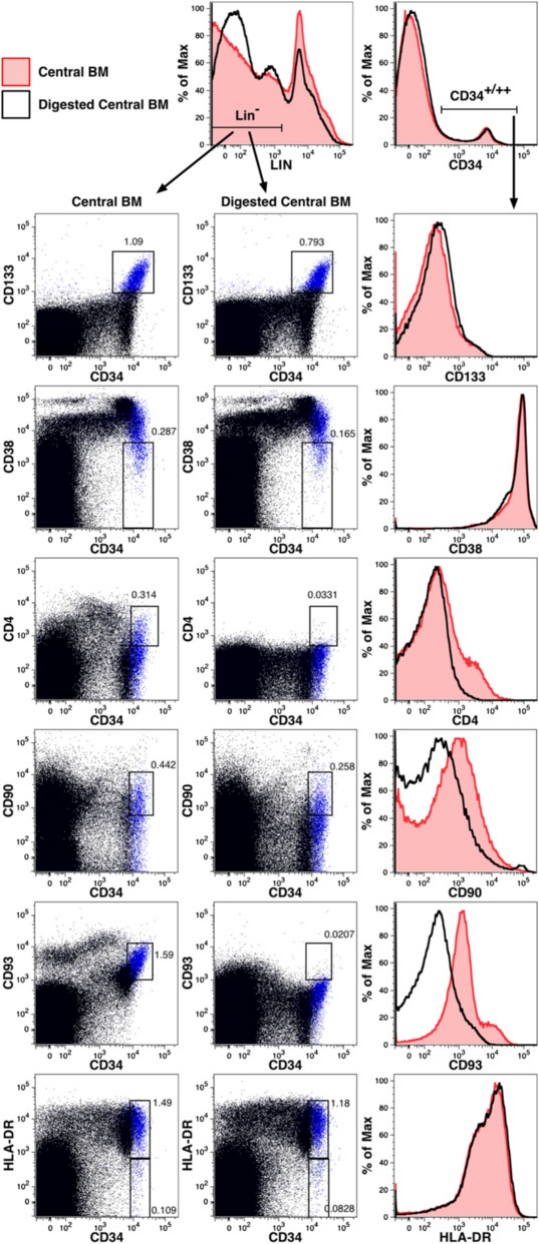
Loss of some cell-surface markers on hematopoietic precursors owing to enzymatic digestion. Flow cytometric analysis was performed on cBM before and after enzymatic digestion was performed as for the isolation of eBM. The data shown in the dot plots are gated on live, single, low side-light scatter cells (not shown) as well as Lin^−^ cells gated as indicated. Expression of CD133 is indicated using blue dots in all dot plots. Numbers represent the frequencies of Lin^−^ cells represented by the gated populations. Overlay histogram plots show the effects of enzyme digestion on antigen expression. The expression of Lin antigens and CD34 are shown on live, single cells (gating not shown), whereas expression of the remaining antigens are shown on live, single cells expressing CD34 as indicated. BM cells were obtained from a 20 weeks’ gestation fetus

Two cell-surface markers associated with hematopoietic stem cells, CD4 and CD93, were notably absent among eBM cells in contrast to the other markers that were studied (Fig. 6). Since all the gated populations shown in Fig. 6 are largely overlapping populations of primitive hematopoietic cells, the lack of CD4 and CD93 expression was difficult to explain in light of the expression patterns of the other HSC markers. We therefore tested if these antigens were susceptible to enzymatic removal by digestion of cBM under the conditions used to isolate the eBM cells. The panel of Lin markers, used to mark mature cell populations, was modestly affected by enzymatic digestions, whereas CD34 expression was not diminished (Fig. 7). Among the markers used to define hematopoietic stem cells and primitive progenitors, CD4 and CD93 were notably eliminated and CD90 was partially reduced. The other markers were not appreciably affected. Note that the loss of Lin antigen, resulting from enzymatic digestion, did reduce the frequency of gated progenitor populations owing to the greater dilution of these populations by cells that would otherwise be Lin^+^ cells.

### Human precursors in the cBM and eBM cells of humanized mice

**Fig. 8:**
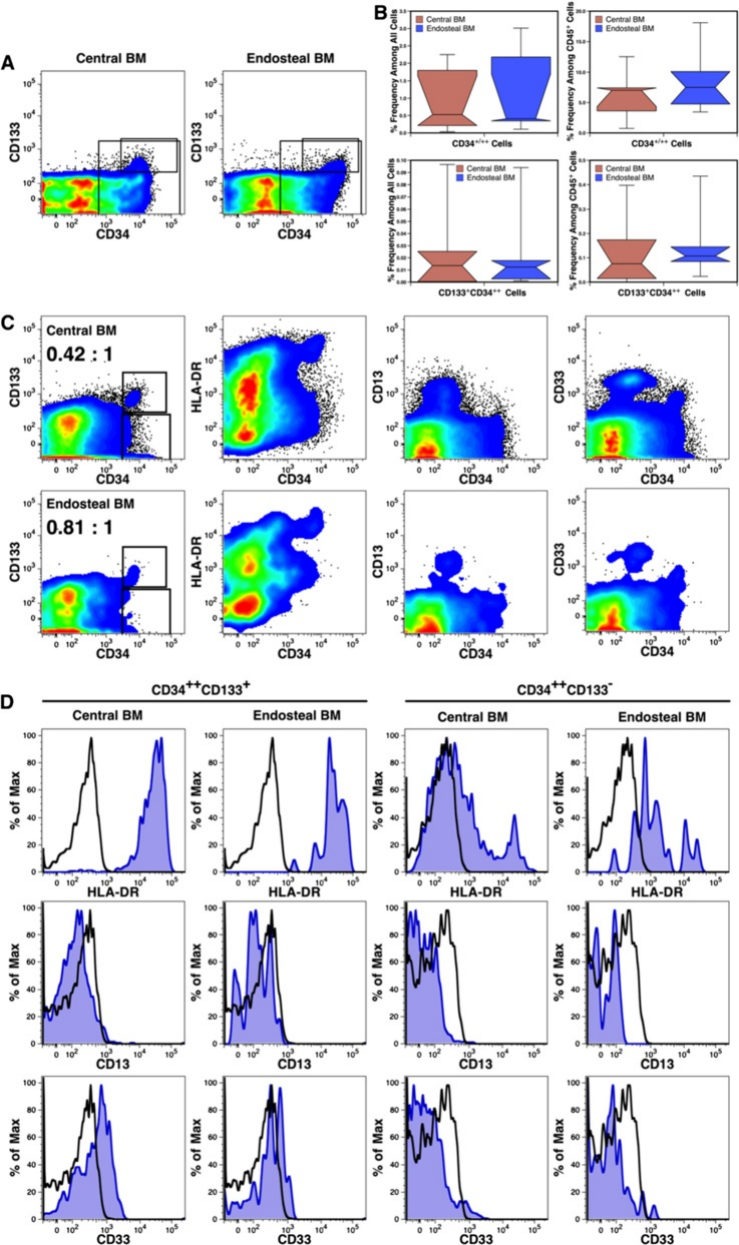
Engraftment of the cBM and eBM by human cells in immunodeficient mice. uPA-NOG mice analyzed 308 days after transplant showed engraftment by CD34^+/++^ (large rectangular region) and CD133^+^CD34^++^ (smaller gated subset) cells in both the cBM and eBM (**a**). The frequencies of these precursor populations found among all live, single cells and human CD45^+^ cells are shown using box plots (n = 5) (**b**). Engraftment of various HSC compartments in irradiated NSG mice is indicated (**c**). Rectangular regions define CD34^++^CD133^+^ and CD34^++^CD133^−^ precursors in the cBM (top row) and eBM (bottom row). The ratio of gated CD133^+^ to CD133^−^ cells are indicated for cBM and eBM. The expression HLA-DR, CD13 and CD33 on CD34^++^CD133^+^ and CD34^++^CD133^−^ precursors from cBM and eBM is shown in histogram plots (**d**). Staining with the indicated antibody is shown with blue shading, whereas staining with an isotype-matched control antibody is shown in black outline

Humanized mice provide a model for studying human hematopoiesis in an in vivo setting [31]. We evaluated cBM and eBM engraftment by human hematopoietic precursors in uPA-NOG mice. In the first experiment, mice were transplanted with hematopoietic precursors isolated from fetal liver, which is the source of HSCs that seed the BM during development. The first cohort of 5 mice were transplanted without irradiation to avoid the cytoablative treatment that may damage the BM and affect the seeding of the cBM and eBM. Although engraftment varied widely, as is typical of humanized mice, precursors expressing CD34 and CD133 were observed among both the cBM and eBM (Fig. 8a). The frequencies of human hematopoietic precursors are shown among all cBM and eBM cells as well as among only the fraction of human CD45^+^ leukocytes found in the cBM and eBM (Fig. 8b). The median percentage of CD34^+/++^ and CD133^+^CD34^++^ cells were modestly higher in the eBM, but the increase was not significant given the range of data from different chimeric mice.

Findings with the unirradiated uPA-NOG mice were followed by transplanting NSG mice, after cytoablative irradiation, with human 24 weeks’ gestation cBM cells. Engraftment of both the cBM and eBM by CD133^+^CD34^++^ cells was observed in two mice analyzed 356 days after transplant (Fig. 8c).

The phenotype of fetal HSCs differs somewhat from that of adult HSCs. We hypothesized that long-term engraftment of human fetal cells in the adult environment of immunodeficient mice would result in a switch from a fetal to an adult phenotype. Most fetal HSCs express HLA-DR, CD13 and CD33 in contrast to their adult counterparts [26, 27]. We examined the expression of these antigens after long-term engraftment in mice to determine if the expression of these markers was lost as on adult cells. HLA-DR, CD13 and CD33 were expressed on cBM and eBM cells (Fig. 8c). In Fig. 8d, expression was also compared on enriched HSCs (CD133^+^CD34^++^) and a population of early-stage committed-progenitors (CD133^−^CD34^++^). HLA-DR was strongly expressed on cells expressing CD133, but had declined on CD133^−^CD34^++^ cells. The most notable difference found on CD34^++^ precursors from cBM and eBM was a shift towards higher HLA-DR expression on CD133^−^ from the eBM compared to the cBM. CD13 expression was not detected on any CD34^++^ precursors, whereas a very low level of CD33 expression was observed on CD133^+^ cells but not CD133^−^ cells. These data indicate that the fetal HSC phenotype has only partially converted to an adult phenotype after nearly 1 year in vivo.

## Discussion

Mice are among the most commonly used laboratory animals in research and the collection of BM from these animals has become a matter of routine. However, somewhat surprisingly, protocols used for the isolation of BM can vary notably from the very simple single-cut method used in this study to the more cumbersome double-cut methods employing varying degrees of soft-tissue removal. This study was performed, in part, to determine if these two methods are comparable in their yield of cBM cells, which they were for harvesting femoral cBM. In the case of the tibia, the single-cut method was found to be superior for maximizing the number of cells that can be harvested. The single-cut method was also notably faster due to its simplicity. Also important, the single-cut method was no more difficult to learn for novice investigators than the double-cut method despite the fact that the latter procedure provides greater visualization of the bone prior to marrow harvest.

A complete harvest of BM is often a requirement for many experiments, either to yield as many cells as possible for analysis or to perform a quantitative analysis of the BM compartment. The higher efficiency of the single-cut method makes it the preferable method to harvest the maximum number of cBM cells. However, the distribution of cell types within the BM is not homogeneous and, consequently, different harvest techniques may vary in their efficiency in isolating particular cell lineages [14]. Our own experience with the single-cut method shows this method to effectively isolate hematopoietic progenitors and long-term reconstituting HSCs [32, 33]. Nonetheless, studies of the HSC niche have shown different types of HSCs and progenitors to reside in different parts of the long-BM. Lord and Hendry showed an increased density of hematopoietic precursors with distance away from the central axis of the bone [15]. Accordingly, higher levels of precursor proliferation are found near the inner wall of the bone, closer to the endosteum [16]. More recently, Haylock et al. and Grassinger et al. demonstrated that phenotypically-defined HSCs were enriched within the eBM and that the HSCs from eBM have greater proliferative and engraftment potential when compared to those from cBM [17, 20]. These authors estimated that about a third of LSK cells and a quarter of CD48^−^CD150^+^ LSK cells reside in the endosteum. Our results yielded somewhat higher recoveries of 49 % of LSK cells and 39 % of CD48^−^CD150^+^ LSK cells from the eBM. The higher yields are likely due to subtle differences in methods, techniques and the bones used to isolate cBM and eBM. On of these differences could be the size of the needle used to isolate the cBM. We found 23–27 gauge needles to be similarly effective in harvesting cBM, but would recommend 23 gauge needles to maximize the recovery of cBM and minimize the quantity of eBM remaining in the marrow. Nonetheless, a complete harvest of HSCs requires harvest of the eBM.

Our study also reveals the value of harvesting tibial BM to increase the overall recovery of BM cells. Data on two common strains of mice show that the tibia can provide an additional 54 % of the number of cells collected from the femur alone. Thus, harvesting the tibia is a good option for increasing the yield of BM cells with minimal extra time required for the collection. The quantity of BM in different long bones has been measured by a number of investigators using mice pulsed with ^59^Fe to radioactively label developing erythroid cells (Table 2). An average femoral BM content from six studies is 6.5 % of total skeletal BM [5, 34–38]. We found only one reported value for tibial BM measured at 3.5 % [34]. However, three other studies measured BM content of both the tibia and fibula at an average 3.1 % [5, 36, 38]. Since the tibia contains nearly all the BM of these two bones, we consider it reasonable to average the tibial measurement made by Chervenick et al. with the other three reported measurements; the average estimate from four studies for tibial BM content is 3.2 %. Based on our calculated averages, harvest of a single femur and tibia should yield 9.7 % of the total skeletal BM (19.4 % for both hind limbs). This estimate is in close agreement with total hind limb measurements made by Boggs and Patrene ranging from 8.4 to 10.75 % [39]. These combined BM measurements are also in agreement with our measurement of an approximate 2:1 ratio of cell content in the femur and tibia.

**Table 2:** Summary of hind limb BM content estimates by ^59^Fe distribution experiments

Study	Single femur	Single tibia	Single tibia + fibula	Single hind leg	Reference
Chervenick et al.	5.9 %	3.5 %	N.D.^a^	N.D.	[34]
Schofield & Cole	7.0 %	N.D.	N.D.	N.D.	[35]
Briganti et al.	6.1 %	N.D.	3.35 %	N.D.	[36]
Papayannopoulou & Finch	7.4 %	N.D.	N.D.	N.D.	[37]
Lee et al.	6.1 %	N.D.	3.05 %	N.D.	[38]
Boggs et al.	6.7 %	N.D.	2.85 %	N.D.	[5]
Boggs & Patrene	N.D.	N.D.	N.D.	8.4 % & 10.75 %^b^	[39]
Average:	6.5 %	3.5 %	3.1 %	9.6 %	

^a^N.D. = Not Determined

^b^Two different methods of preparation were performed yielding modestly different results

We evaluated the benefit of enzymatic digestion to isolate fetal human eBM cells. Although the method used for enzymatic digestion of the human BM was that same as for the murine samples, it is important to note that the isolation of human cBM by flushing was prone to more variability. Unlike the single-cut method where insertion of the needle into the BM cavity leaves little room for variability, flushing of the larger human bones requires multiple needle-point entries and physical crushing of the bones to access the cBM throughout the marrow cavity. A simple visual goal employed in cBM isolation was to flush most of the red cells from the bones. Nonetheless, the amount of tissue processed, the size of the bone and the gestational age of the sample can all affect the harvest. We observed eBM to comprise a consistent minimum of 7 % of the total BM content.

We observed an increased frequency of phenotypically defined HSCs in the eBM preparations made from fetal long bones. These results are in agreement with the reported enrichment of HSCs in the trabecular bone area of adult posterior ileac crest by Guezguez et al. [18]. These authors observed a higher frequency of Lin^−^CD34^++^CD38^−^CD45RA^−^CD49f^+^ cells in the trabecular bone area, found near the surface of the bone biopsy, compared to the long bone area, defined as being closer to the central core of the biopsy. We observed HSC subsets such as CD34^++^CD133^+^, CD34^++^CD38^low^, CD34^++^CD90^+^ and CD34^++^HLA-DR^+^ cells enriched among eBM cells. It was also noted that the enzymatic digestion used to isolate the eBM cells reduced CD4, CD90 and CD93 antigen expression and, consequently, HSCs are best identified using antigens less prone to enzymatic digestion such as CD133, CD38 and HLA-DR.

Human fetal tissue obtained from elective abortions has been used as a source of donor tissue for transplantation and banks of fetal tissue have been established to accommodate such efforts [40–42]. Many of these efforts have centered around the use of fetal donor cells for prenatal transplantation where the patient is also a fetus, thereby matching the developmental stage of the donor cells and the host. Although the fetal liver has been most studied and used as a source of HSCs for transplantation, fetal BM has also been evaluated as a source of HSCs for transplantation [12]. The yield of cells available from the long bones varies significantly with gestational age and only by mid-gestation do these tissues offer an abundant source of hematopoietic cells. At 20 weeks’ gestation, we recovered 1.9 × 10^6^ CD34^++^CD133^+^ and 2.5 × 10^5^ CD34^++^CD38^low^ cells from the harvest of all long bones, with 10-20 % of the cells coming from the eBM preparation. These values are comparable to the yields of cells from umbilical cord blood [43, 44]. HSC recoveries from fetal BM obtained at 23–24 weeks’ gestation can exceed those of a typical umbilical cord blood harvest as demonstrated in this study and previously by Golfier et al. [12]. The increased yield of HSCs offered by harvesting eBM as well as the greater proliferative and engraftment potential of eBM HSCs clearly favors the harvest of eBM for any clinical application.

Humanized mice were created by two methods to test the engraftment of the eBM compartment in mice by human HSCs. In the first model, intra-splenic injection was used in mice that had not been preconditioned with irradiation to preserve the integrity of the normal BM environment. These mice were also transplanted with fetal liver cells, a source of HSCs not compartmentalized into cBM and eBM cells. We observed engraftment of both BM compartments with human donor cells, showing for the first time that human fetal liver cells do engraft the eBM. Similar findings were obtained when mice were transplanted with cBM cells from fetal BM, demonstrating that cBM can engraft the eBM niche. These findings are in agreement with Guezguez et al., who observed that when human cells, engrafted in mice, were isolated from either niche, they could reconstitute both niches in secondary recipients [18]. We have also successfully engrafted human multilineage hematopoiesis in secondary murine hosts using cBM cells isolated by the single-cut method [23]. Despite the capacity of HSCs from cBM to engraft eBM, molecular and functional differences between cBM and eBM have been observed and are believed to be regulated, at least in part, by the differences in the cBM and eBM niches [18].

The phenotypic analysis of fetal human hematopoietic cells engrafted in mice also offered the opportunity to evaluate the expression of several antigens expressed on HSCs only early in ontogeny. We were interested to determine if the fetal HSC phenotype changes to an adult phenotype when cells are transplanted into an adult mouse and analyzed at an age corresponding to about 8 months after a normal term delivery. CD13 and CD33 are generally described as myeloid differentiation antigens that are not expressed on adult stem cells [45–51], but these antigens are observed on fetal HSCs [26, 27]. Moreover, CD33 expression on fetal peripheral blood HSCs (CD34^+^CD38^−^ and CD34^+^CD117^+^) has been shown to decline with gestational age, with its expression near absent at term birth [52]. Herein, we show that CD13 expression is lost from fetal HSCs maintained in NSG mice, and that CD33 expression also has declined but low level expression remains. The expression of these markers was similar for cBM and eBM. These data show at least a partial shift towards the adult HSC phenotype. HLA-DR expression remained high on engrafted CD133^+^ HSCs, however, resembling the expression observed in midgestation fetal liver [26, 27]. There was also a shift towards more HLA-DR expression among early progenitors (CD34^++^CD133^−^) in the eBM than in the cBM. These findings are in contrast to adult cells for which evidence indicates that the most primitive hematopoietic precursors lack HLA-DR expression [29, 53–55]. Thus, based on HLA-DR expression, transplanted fetal HSCs do not appear to fully acquire an adult phenotype after long-term reconstitution in immunodeficient mice. It should be noted, however, that unavailability of data on the HSC phenotype in children leaves open the possibility that the human HSC phenotype observed in our engrafted mice is actually representative of a hypothetical transitional phenotype that may be observed in neonates and young children.

## Conclusions

For most experiments requiring mouse BM cells, the single-cut method offers the simplest, most efficient and fastest method of BM isolation from the femur and tibia. The isolation of HSCs, however, is more complete using enzymatic digestion to harvest the eBM. Enzymatic digestion of human BM also increases the yield of cells and HSCs, but the effects of the digestion on cell-surface antigen expression must be tested to avoid false-negative results. Humanized mice offer an in vivo model to study trafficking of human HSC to the cBM and eBM niches with changes in phenotype noted as fetal HSCs aged in mice.

## Additional file

Additional file 1:
**Video demonstrating the single-cut method of cBM isolation.**

## Abbreviations

AF700: Alexa Fluor 700
APC: Allophycocyanin
APC-Cy7: Allophycocyanin-cyanine 7
BM: Bone marrow
cBM: Central bone marrow
eBM: Endosteal bone marrow
FITC: Fluorescein isothiocyanate
HSC: Hematopoietic stem cell
Lin: Lineage
LSK: Lin^−^SCA-1^+^CD117^+^
MSC: Mesenchymal stromal cell
NSG: NOD.Cg-*Prkdc*^*scid*^*Il2rg*^*tm1Wjl*^/SzJ
PB: Pacific blue
PBS: Phosphate-buffered saline
PE: Phycoerythrin
PE-Cy7: Phycoerythrin-cyanine 7
uPA-NOG: NOD.Cg-*Prkdc*^*scid*^*Il2rg*^*tm1Sug*^*Tg*(*Alb*-*Plau*)*11*-*4*/ ShiJic
